# Identification of a Breast Cancer Susceptibility Locus at 4q31.22 Using a Genome-Wide Association Study Paradigm

**DOI:** 10.1371/journal.pone.0062550

**Published:** 2013-05-22

**Authors:** Yadav Sapkota, Yutaka Yasui, Raymond Lai, Malinee Sridharan, Paula J. Robson, Carol E. Cass, John R. Mackey, Sambasivarao Damaraju

**Affiliations:** 1 Cross Cancer Institute, Edmonton, Alberta, Canada; 2 Department of Laboratory Medicine and Pathology, University of Alberta, Edmonton, Alberta, Canada; 3 School of Public Health, University of Alberta, Edmonton, Alberta, Canada; 4 Department of Agricultural, Food and Nutritional Sciences, University of Alberta, Edmonton, Alberta, Canada; 5 Department of Oncology, University of Alberta, Edmonton, Alberta, Canada; 6 Alberta Health Services – Cancer Care, Edmonton, Alberta, Canada; IFOM, Fondazione Istituto FIRC di Oncologia Molecolare, Italy

## Abstract

More than 40 single nucleotide polymorphisms (SNPs) for breast cancer susceptibility were identified by genome-wide association studies (GWASs). However, additional SNPs likely contribute to breast cancer susceptibility and overall genetic risk, prompting this investigation for additional variants. Six putative breast cancer susceptibility SNPs identified in a two-stage GWAS that we reported earlier were replicated in a follow-up stage 3 study using an independent set of breast cancer cases and controls from Canada, with an overall cumulative sample size of 7,219 subjects across all three stages. The study design also encompassed the 11 variants from GWASs previously reported by various consortia between the years 2007–2009 to (i) enable comparisons of effect sizes, and (ii) identify putative prognostic variants across studies. All SNP associations reported with breast cancer were also adjusted for body mass index (BMI). We report a strong association with 4q31.22-rs1429142 (combined per allele odds ratio and 95% confidence interval = 1.28 [1.17–1.41] and *P*_combined_ = 1.5×10^−7^), when adjusted for BMI. Ten of the 11 breast cancer susceptibility loci reported by consortia also showed associations in our predominantly Caucasian study population, and the associations were independent of BMI; four *FGFR2* SNPs and *TNRC9*-rs3803662 were among the most notable associations. Since the original report by Garcia-Closas et al. 2008, this is the second study to confirm the association of 8q24.21-rs13281615 with breast cancer outcomes.

## Introduction

Breast cancer is the most common cancer in women in the developed world, with 22,700 new cases and 5,100 deaths anticipated in Canada for 2012 [1]. While environmental and lifestyle risk factors contribute to most of the variation in breast cancer risk, twin studies have shown substantial contribution of inherited genetic risk factors to disease susceptibility [2], [3]. Linkage and family-based studies have identified high and moderate penetrance mutations in genes such as *BRCA1* [4], *BRCA2* [5], *PTEN* [6], *ATM* [7], *TP53* [8], *BRIP1* [9], *PALB2* [10] and *CHEK2* [11] contributing to hereditary breast cancer; however, these mutations occur rarely in the general population. Further, linkage studies failed to identify additional genes/mutations associated with high or moderate risk of breast cancer. Therefore, it has been hypothesized that most of the genetic risk of breast cancer, for both familial and sporadic cases in the general population, may involve a combination of multiple low penetrance genes/loci, each contributing to an overall genetic risk of breast cancer [12].

Over the past five years, several genome-wide association studies (GWASs) reported breast cancer susceptibility variants (*i.e.*, single nucleotide polymorphisms, SNPs) at multiple loci [13]–[22]. A large-scale candidate gene study also identified an additional locus (caspase 8 coding SNP, rs1045485) associated with breast cancer risk [23]. The low penetrance common SNPs identified to date explain less than 10% of the genetic risk of breast cancer [22]. Taken together, pathogenic germline mutations and low penetrance variants identified thus far only account for a small fraction of the genetic risk of breast cancer, suggesting that additional variants remain to be identified [24].

Recently, we conducted a two-stage GWAS using sporadic breast cancer cases and apparently healthy controls and identified six SNPs (located at chromosomes 4, 5, 16 and 19) that appeared to be associated with breast cancer susceptibility [21]. In a combined sample size of 1,455 breast cancer cases and 1,536 healthy controls from two independent stages, these SNPs showed modest risk of breast cancer (observed odds ratios (ORs) range: 1.22–1.45).

It is an internationally accepted practice to replicate GWAS findings in multiple independent studies with cases and controls of both similar and diverse ethnic backgrounds to assess the robustness and generalizability of the identified associations, respectively. Therefore, in the current study, we further investigated the six putative breast cancer susceptibility SNPs that we have reported previously [21] by conducting an independent replication study (stage 3), using breast cancer cases and controls. The study subjects were predominantly of Caucasian origins drawn from the same geographical region in Canada as in our previous study. Further, we evaluated the GWAS variants for breast cancer susceptibility reported by various consortia. These include the Breast Cancer Association Consortium [13], [18], the Effectiveness of Additional Reductions in Cholesterol and Homocysteine Collaborative Group [13], the Nurses' Health Study [14], the National Cancer Institute Cancer Genetic Markers of Susceptibility Project [14] and the National Heart, Lung, and Blood Institute Framingham Heart Study [15]. We compared the consortia reported variants with our study population to explore the extent of conformity to previous findings within the Caucasian populations, and for the strengths of associations for the sample size utilized in this study. Since obesity is a well-established risk factor for post-menopausal breast cancer [25] and is a heritable trait [26], we also adjusted the identified variant-breast cancer associations for body mass index (BMI) to examine whether the variants are associated with breast cancer risk, through BMI or through different pathways. We assessed variability in disease susceptibility by clinicopathological characteristics such as menopausal status, family history of breast cancer, luminal A status of tumors, tumor grade and tumor stage. Finally, we explored the associations of the six putative susceptibility SNPs identified in our earlier study and the previously published consortia SNPs with breast cancer outcomes.

## Materials and Methods

### Study participants

All breast cancer cases (n = 2,750) used in this study had a confirmed diagnosis of breast cancer in the province of Alberta, Canada, and participated in provincial tumor bank projects in operation since 2001 (the PolyomX Project, 2001–2005 and subsequently merged with the Canadian Breast Cancer Foundation (CBCF) Tumor Bank, 2005 to present; http://www.abtumorbank.com/), Alberta, Canada) [21], [27]. The tumor bank accrues tumor tissues and blood samples from patients with confirmed diagnoses of breast and other cancer types, through hospitals (publicly funded comprehensive cancer care centres managed by the Alberta Health Services (AHS)) in Edmonton and Calgary in the province of Alberta, Canada. The tumor bank database contains well-annotated clinicopathological information for the banked specimens. The CBCF Tumor Bank currently holds blood from more than 8,000 individuals from various cancer types, as a source of germline DNA for genotyping, in addition to tumor tissue specimens. Apparently-healthy (*i.e.*, confirmed not to have had a diagnosis of any cancer) controls (n = 4,472) were obtained from the Tomorrow Project (http://in4tomorrow.ca) and were frequency matched to cases based on ten-year age group. The Tomorrow Project is a large prospective cohort study that started in 2000 and successfully recruited approximately 42,000 Albertans (64% women) by 2012 using a combination of random digit dialling (RDD), and random mail-outs, augmented by email campaigns and social media. Inclusion criteria for initial recruitment to the Tomorrow Project were as follows: (i) aged 35–69 years; (ii) no personal history of cancer, other than non-melanoma skin cancer; (iii) able to complete written questionnaires in English and (iv) currently living in Alberta. Upon enrolment to the Tomorrow Project, participants completed a health and lifestyle questionnaire (including family history of major diseases). The participants gave written consent to be contacted in the future to provide a blood sample for banking to support research in cancer or chronic diseases, receive invitations to provide updated health and lifestyle information or additional samples in the future, and to linkage with administrative health data to understand patterns of health services utilization and disease occurrence [28]. Absence of prior history of cancer upon study enrolment was confirmed by performing linkage with the Alberta Cancer Registry (http://www.albertahealthservices.ca/poph/hi-poph-surv-cancer-alta-cancer-registry-2009.pdf). As of late 2012, approximately 19,000 Tomorrow Project participants from across Alberta had given a 50 ml non-fasting venous blood sample for banking in multiple aliquots of buffy coat, serum, plasma and red blood cells. Breast cancer cases in this study were of predominantly Caucasian ancestry, and resided in the Edmonton and Calgary regions (sites of tertiary cancer centres in Alberta). The population in these regions accounts for two thirds of the total population of the province of Alberta. Thus, in addition to age matching, the controls were selected from the Tomorrow Project using the same ethnicity and geographic location criteria. Even though socio-economic status (SES) plays a role in health outcomes, differences between SES of cases and controls used in this study and underlying assumptions needs to be validated independently. However, given the universal access to health care as a model adopted in Canada, the influence of SES was therefore considered as minimal, if any. A brief description of demographic characteristics of breast cancer cases and controls is presented in **Table 1**. Written informed consent to use banked samples to support research was obtained from all the study participants, and the study was approved by the Alberta Cancer Research Ethics Committee, Alberta, Canada.

**Table 1:** Distribution of age and BMI of breast cancer cases and controls used in the study.

Characteristics	Breast cancer cases (n = 2750)	Apparently healthy controls (n = 4472)
Median age in years at diagnosis/blood draw [range]	54 [22–92]	54 [35–78]
<40	192	343
40–50	710	1282
50–60	889	1538
60–70	635	1144
70–80	242	162
>80	64	0
Missing	18	3
Median body mass index (kg/m^2^) [25th–75th percentiles]	27.4 [24.1–31.4]	25.5 [22.7–29.3]
<18.5	20	41
18.5–24.99	663	1899
25–39.99	1359	2155
>40	112	148
Missing	596	229

### SNPs and samples used

In this replication study (stage 3), we investigated associations of the six putative breast cancer susceptibility SNPs (4q31.22-rs1429142, 5p15.2-rs1092913, 16q23.2-rs1981867, *ZNF577*-rs10411161, *ZNF577*-rs3848562 and *ZNF577*-rs11878583) [21], that we reported in our previous two-stage GWAS. Stage 3 (total n = 4,228) of the study used an independent set of breast cancer cases (n = 1,294) and healthy controls (n = 2,934). In the combined analyses of all three stages, a cumulative sample size (total n = 7,219) was used. We also assessed the strengths of 11 breast cancer susceptibility SNPs that had been reported by consortia until 2009 (*SLC4A7*-rs4973768 [18], 5p12-rs4415084 [16], 5p12-rs10941679 [16], 5q11.2-rs889312 [13], 8q24.21-rs13281615 [13], *FGFR2*-rs2981579 [19], *FGFR2*-rs1219648 [14], *FGFR2*-rs2420946 [14], *FGFR2*-rs2981582 [13], *TNRC9*-rs3803662 [13] and *COL1A1*-rs2075555 [15]). A cumulative sample size of 2,672 breast cancer cases and 4,470 apparently healthy controls were genotyped for these 11 consortia SNPs. Genotype data are available upon request.

### SNPs genotyping and quality control

Germline DNA was extracted from peripheral blood samples of both cases and controls using commercially available Qiagen (Mississauga, ON, Canada) DNA isolation kits. All genotyping assays were performed on the Sequenom iPLEX Gold platform (San Diego, CA, USA) using services from the McGill University and Genome Quebec Innovation Center, Montreal, Canada. Within-stage (stage 3 for the six SNPs from our previous GWAS and a single stage for the 11 consortia SNPs) genotype concordance was assessed with 66 duplicate samples (8 cases and 58 controls). Cross platform (Affymetrix vs. Sequenom *i.e.*, stage 1 vs. stage 3 for the six SNPs) was assessed with 17 duplicate samples (5 cases and 12 controls). Between-stage (stage 2 vs. stage 3 for the six SNPs) genotype concordance was assessed with 632 cases and 452 controls. Duplicate samples used for assessing genotype concordances among various stages were randomly selected. Very stringent criteria of SNP call rate >99% was considered to minimize false positive associations due to missing genotype counts and HWE criteria of *P*>10^−6^ in control subjects were adopted.

### Association analyses and statistical considerations

#### Overall analyses

Allelic associations of SNPs with breast cancer susceptibility were evaluated with correlation/trend tests with one degree of freedom (d.f.). The strengths of allelic and genotypic associations were estimated using unconditional logistic regressions and reported as ORs and 95% confidence intervals (CIs). To increase sample size and hence the statistical power to better capture SNP-breast cancer associations, cases and controls from all independent stages were pooled together and combined analyses were conducted. BMI was included as a covariate in the logistic models to calculate adjusted ORs, 95% CIs and *P* values in Stage 3 and in combined stages.

#### Subgroup analyses

To evaluate variations in SNP-breast cancer associations by clinicopathological characteristics (to address potential heterogeneity in the observed overall associations), we conducted subgroup analyses (unconditional logistic regressions adjusted for BMI) within the combined breast cancer cases based on menopausal status, luminal A status, family history of breast cancer (captured under the single category representing cases with first, second or third degree relatives), tumor stage and grade. A common set of healthy controls was used to test the SNP-breast cancer associations in these subgroup analyses. Breast tumors that were either estrogen receptor (ER) or progesterone receptor (PR) positive and human epidermal growth factor receptor 2 (HER2) negative were classified as luminal As, and the remainder were classified as non-luminal As. The cases with unknown ER, PR or HER2 status were excluded from the luminal A subgroup analyses. Breast tumors with operable tumor stages (I-IIIA) were classified as one subgroup while tumors with non-operable tumor stages (IIIB, IIIC) were classified as the other subgroup. Heterogeneity in ORs between the subgroups was assessed using multinomial logistic regressions (‘*mlogit*’) and linear combination of estimators (‘*lincom*’) implemented in Stata 12.0 (www.stata.com). Statistical significance of this heterogeneity test was reported as *P* for heterogeneity (*P*_het_).

#### Associations of SNPs with breast cancer outcomes

We also evaluated the potential prognostic values of SNPs with breast cancer outcomes, such as recurrence-free survival (RFS) and overall survival (OS), by fitting Cox proportional hazards models available in the “survival” package [29] implemented in R 2.15.1 [30], adjusted for BMI. The associations were reported as hazard ratios (HRs), 95% CIs and adjusted *P* values. Genotypes were recoded to 0 (wild type homozygotes), 1 (heterozygotes) and 2 (variant homozygotes) before fitting the Cox models.

All statistical tests were two-sided. We assumed an additive model of genetic inheritance to calculate power, as described earlier [21]. As such, our study had adequate power (>80%) to detect associations that were larger than genotypic relative risk of ≥1.2. Whenever multiple SNPs were tested, correction for multiple hypotheses testing was performed by *P* = 0.05/number of tests. We considered all SNPs from our stage 1 GWAS (782,838 SNPs) to calculate genome-wide significance (*P*<6.4×10^−8^) for the six replicated SNPs. Correlation/trend tests were carried out using SNP and Variation Suite v7.6.11 (Golden Helix, Inc., Bozeman, MT, www.goldenhelix.com) [31]. The observed and adjusted allelic and genotypic ORs and 95% CIs and adjusted *P* values were estimated using logistic models in PLINK (http://pngu.mgh.harvard.edu/~purcell/plink/) [32]. All the general statistical analyses were conducted using R 2.15.1.

## Results

Genotyping assays of the 17 SNPs considered in this study were successful with a SNP call rate of >99%. Average within-stage genotype concordance was 100% while cross-platform genotype concordance was >99%; between-stage average genotype concordance was also 100%. We reasoned that this negligible percentage (<1%) of discordance was unlikely to influence SNP-breast cancer associations and hence all the genotype data were considered for the downstream association analyses. The genotype distributions from the six SNPs (our previous work) showed conformity with Hardy-Weinberg Equilibrium (HWE) criteria in control subjects. Similarly, the genotype distributions from the 11 consortia SNPs were also in agreement with HWE. Minor allele frequencies (MAFs) of the six SNPs across all stages and the 11 consortia SNPs were comparable with the published MAFs, reflecting the robustness of the genotyping platform vis-à-vis negligible genotyping errors (**Tables 2** **and S1**) and confidence in the reported associations.

**Table 2:** Replication of the six putative breast cancer susceptibility loci in independent stage 3.

									Observed				Adjusted***			
SNP	Genes/loci	Cytoband	Location (bp)*	MA	Stage#	MAF	Call rate	Cases/controls	OR_per-allele_ [95% CI]	OR_heterozygote_ [95% CI]	OR_homozygote_ [95% CI]	*P*_corr/trend_ **	OR_per-allele_ [95% CI]	OR_heterozygote_ [95% CI]	OR_homozygote_ [95% CI]	*P*
rs1429142	*EDNRA*/Intergenic	4q31.22	148,289,389	C	1	0.17	1.00	302/321	1.55 [1.15–2.08]	1.51 [1.06–2.15]	2.65 [1.06–6.63]	2.82E-03	ND			
					2	0.18	1.00	1153/1215	1.21 [1.04–1.40]	1.25 [1.05–1.49]	1.29 [0.82–2.04]	1.28E-02	ND			
					3	0.18	1.00	1294/2934	1.20 [1.07–1.34]	1.23 [1.06–1.42]	1.33 [0.94–1.86]	2.50E-03	1.23 [1.08–1.40]	1.28 [1.09–1.50]	1.34 [0.92–1.95]	1.70E-03
					1+2+3	0.18	1.00	2749/4470	1.22 [1.12–1.33]	1.26 [1.13–1.40]	1.36 [1.06–1.75]	4.71E-06	1.28 [1.17–1.41]	1.32 [1.17–1.48]	1.52 [1.16–2.00]	1.50E-07
rs1092913	*ROPN1L*/Intergenic	5p15.2	10,467,702	T	1	0.10	1.00	302/321	1.89 [1.29–2.76]	2.24 [1.46–3.44]	1.23 [0.30–4.97]	7.03E-04	ND			
					2	0.14	0.99	1153/1215	1.34 [1.14–1.57]	1.27 [1.04–1.55]	2.10 [1.27–3.49]	2.17E-04	ND			
					3	0.14	1.00	1294/2934	1.15 [1.01–1.30]	1.07 [0.91–1.26]	1.57 [1.10–2.24]	3.14E-02	1.17 [1.02–1.34]	1.07 [0.89–1.28]	1.70 [1.16–2.50]	2.89E-02
					1+2+3	0.13	0.99	2749/4470	1.22 [1.11–1.34]	1.19 [1.06–1.34]	1.62 [1.23–2.14]	2.18E-05	1.21 [1.10–1.34]	1.20 [1.05–1.36]	1.53 [1.13–2.06]	1.96E-04
rs1981867	*C16orf61*/Intergenic	16q23.2	80,923,769	T	1	0.29	1.00	302/321	1.59 [1.23–2.05]	1.50 [1.08–2.08]	2.82 [1.49–5.34]	3.72E-04	ND			
					2	0.31	1.00	1153/1215	1.14 [1.01–1.29]	1.00 [0.84–1.18]	1.53 [1.15–2.03]	3.17E-02	ND			
					3	0.32	1.00	1294/2934	0.97 [0.88–1.08]	1.00 [0.87–1.14]	0.92 [0.73–1.16]	6.04E-01	0.99 [0.89–1.11]	1.04 [0.90–1.22]	0.92 [0.71–1.19]	8.46E-01
					1+2+3	0.31	1.00	2749/4470	1.07 [0.99–1.15]	1.02 [0.93–1.13]	1.19 [1.01–1.40]	8.60E-02	1.07 [0.98–1.15]	1.06 [0.95–1.18]	1.15 [0.95–1.37]	1.21E-01
rs10411161	*ZNF577*	19q13.41	52,372,976	A	1	0.11	1.00	302/321	1.79 [1.25–2.57]	1.69 [1.13–2.54]	4.80 [1.01–22.83]	1.08E-03	ND			
					2	0.14	0.99	1153/1215	1.28 [1.09–1.49]	1.34 [1.09–1.65]	1.49 [0.99–2.24]	6.17E-04	ND			
					3	0.13	1.00	1294/2934	0.97 [0.84–1.11]	0.98 [0.84–1.15]	0.87 [0.54–1.38]	6.15E-01	1.00 [0.86–1.17]	1.00 [0.83–1.19]	1.03 [0.63–1.70]	9.67E-01
					1+2+3	0.13	1.00	2749/4470	1.13 [1.03–1.24]	1.11 [0.99–1.25]	1.36 [1.02–1.81]	1.06E-02	1.13 [1.02–1.25]	1.11 [0.97–1.26]	1.35 [0.99–1.85]	2.10E-02
rs3848562	*ZNF577*	19q13.41	52,379,835	C	1	0.11	1.00	302/321	1.82 [1.27–2.61]	1.72 [1.15–2.58]	4.82 [1.01–22.93]	8.05E-04	ND			
					2	0.13	1.00	1153/1215	1.32 [1.11–1.56]	1.34 [1.10–1.64]	1.62 [0.92–2.85]	9.79E-04	ND			
					3	0.13	1.00	1294/2934	0.96 [0.84–1.10]	0.98 [0.84–1.15]	0.85 [0.54–1.36]	5.91E-01	1.01 [0.87–1.17]	1.00 [0.83–1.19]	1.07 [0.65–1.73]	9.18E-01
					1+2+3	0.13	1.00	2749/4470	1.12 [1.01–1.23]	1.13 [1.01–1.27]	1.19 [0.86–1.65]	2.56E-02	1.13 [1.02–1.26]	1.13 [1.00–1.29]	1.26 [0.89–1.79]	2.60E-02
rs11878583	*ZNF577*	19q13.41	52,388,546	C	1	0.12	1.00	302/321	1.80 [1.25–2.58]	1.65 [1.11–2.45]	8.43 [1.03–69.00]	1.26E-03	ND			
					2	0.13	1.00	1153/1215	1.25 [1.06–1.47]	1.16 [0.95–1.41]	2.15 [1.21–3.83]	7.60E-03	ND			
					3	0.13	1.00	1294/2934	0.98 [0.86–1.12]	0.96 [0.82–1.13]	1.01 [0.66–1.56]	7.40E-01	1.00 [0.86–1.16]	0.96 [0.80–1.14]	1.18 [0.74–1.86]	9.98E-01
					1+2+3	0.13	1.00	2749/4470	1.10 [1.00–1.22]	1.07 [0.95–1.20]	1.38 [1.01–1.89]	4.65E-02	1.11 [0.99–1.23]	1.07 [0.94–1.22]	1.38 [0.98–1.94]	6.44E-02

* National Center for Biotechnology Information genome build 37.

MA, minor allele; MAF, minor allele frequency.

** To maintain the consistency in the statistical test, associations of these SNPs were evaluated with correlation/trend test with one d.f. in this study, unlike chi-squared test in the original study (Sehrawat et al. 2011).

*** Adjusted for BMI.

# Association analyses (correlation/trend tests) of Stages 1 and 2 are reported in Sehrawat et al. 2011. Stage 3 and the combined (1+2+3) stages were conducted in this follow-up study.

### Association of previously identified (consortia SNPs) breast cancer susceptibility loci

Except for *COL1A1*-rs2075555, we successfully replicated the association of ten consortia reported breast cancer susceptibility loci in our study population at *P*<0.05 (**Table S1**). These SNPs remained statistically significant after correction for multiple hypothesis testing (*P*<0.05/11 = 0.004). Four *FGFR2* SNPs and *TNRC9*-rs3803662 showed the strongest associations attaining the commonly adopted genome-wide significance level (*P*<5.0×10^−8^), with similar ORs to the original study findings [13], [14], [19]. After adjusting for BMI, five SNPs remained statistically significant (adjusted *P*<4.2×10^−8^) (**Table S1**). The adjusted per allele ORs and 95% CIs were also similar to the observed ORs and 95% CI (**Table S1**), indicating that these SNP-breast cancer associations are independent of the pathway linking BMI and risk of breast cancer.

### Replication of the six putative SNPs in stage 3 analyses

Of the six putative breast cancer susceptibility SNPs that we reported earlier, 4q31.22-rs1429142 showed consistent reproducibility across all three stages. The variant at 5p15.2-rs1092913 also retained statistical significance for increased breast cancer risk in the current independent replication stage 3 study at *P*<0.05 (**Table 2**), and remained statistically significant after correction for multiple hypothesis testing (*P*<0.05/6 = 0.008). The magnitude and direction of per allele ORs and 95% CIs of both SNPs were consistent with our previous findings [21] while slightly elevated ORs and 95% CIs were observed for heterozygotes and variant homozygotes (**Table 2**), conforming to the additive model of genetic inheritance. After adjustment for BMI, both 4q31.22-rs1429142 and 5p15.2-rs1092913 remained statistically significant at adjusted *P*<0.05, while both adjusted per allele and genotypic ORs and 95% CIs of 4q31.22-rs1429142 were larger than the observed ORs. The remaining four SNPs did not show statistical significance at *P*<0.05 in this stage 3 study.

### Combined analyses of the six putative SNPs (stages 1+2+3)

In the combined analyses (stages 1+2+3), five of the six SNPs were significantly associated with increased breast cancer risk at *P*<0.05, the exception being 16q23.2-rs1981867 which showed marginal statistical significance (*P* = 0.06) (**Table 2**). Again, 4q31.22-rs1429142 and 5p15.2-rs1092913 showed the strongest associations after multiple hypotheses correction. The five SNPs retained statistical significance after adjusting for BMI. Interestingly, 4q31.22-rs1429142 achieved near genome-wide significance level with greater per allele and genotypic ORs and 95% CIs (adjusted *P* = 1.5×10^−7^, adjusted per allele OR and 95% CI = 1.28 [1.17–1.41], adjusted OR_heterozygote_ and 95% CI = 1.32 [1.17–1.48] and adjusted OR_homozygote_ and 95% CI = 1.52 [1.16–2.00]), indicating that the 4q31.22-rs1429142-breast cancer association may be linked to the BMI pathway of breast cancer risk elevation (**Table 2**). 5p15.2-rs1092913 also showed a strong association with breast cancer risk (adjusted *P* = 2.0×10^−4^, adjusted per allele OR and 95% CI = 1.21 [1.10–1.34], adjusted OR_heterozygote_ and 95% = 1.20 [1.05–1.36] and adjusted OR_homozygote_ and 95% CI = 1.53 [1.13–2.06]).

### Subgroup analyses

The previously reported GWAS variants (consortia SNPs), except *COL1A1*-rs2075555, remained statistically significant in subgroups with both pre and postmenopausal women, luminal A cases, cases with or without family history of breast cancer, low tumor grade and operable tumor stage at adjusted *P*<0.05 (**Table S2**). The adjusted per allele ORs, 95% CIs and *P* values were also comparable to the overall analyses, with similar magnitudes and directions of risk (**Tables S1 and S2**). Of these, the four *FGFR2* SNPs retained genome-wide significance level in subgroups with luminal A cases, cases with family history of breast cancer, low tumor grade and operable tumor stage while 8q24.21-rs13281615 and *TNRC9*-rs3803662 showed genome-wide significance level associations only in cases with low tumor grade and operable tumor stage, respectively. *SLC4A7*-rs4973768, 5q11.2-rs889312, 8q24.21-rs13281615 and *TNRC9*-rs3803662 showed marginal associations in subgroup with non-luminal A cases. Similarly, 5q11.2-rs889312 and *TNRC9*-rs3803662 showed significant associations in cases with high tumor grade. None of the SNPs showed significant associations in cases with non-operable tumor stage, with the possible exception of 5q11.2-rs889312 which showed a marginally statistically significant association (adjusted *P* = 0.04).

The associations of the six GWAS-identified putative SNPs from our populations with breast cancer were consistent across the subgroups, without any substantial modifications in SNP-breast cancer associations observed in overall analyses (**Table 2**). 4q31.22-rs1429142 and 5p15.2-rs1092913 remained significantly associated in subgroups with both pre and postmenopausal women, luminal and non-luminal A cases and cases without family history of breast cancer, high and low tumor grades and operable tumor stage at adjusted *P*<0.05 (**Tables 3** **and** **4**). Moreover, 4q31.22-rs1429142 attained genome-wide significance level in subgroups with premenopausal women (adjusted *P* = 6.2×10^−10^), while a strong statistical association was also observed in cases with operable tumor stages (adjusted *P* = 1.6×10^−7^). The *ZNF577* SNPs (rs10411161, rs3848562 and rs11878583) also showed statistically significant associations in subgroups with postmenopausal women, luminal A cases, cases without family history and operable tumor stages (**Tables 3** **and** **4**).

**Table 3:** Subgroup analyses of the six putative breast cancer susceptibility SNPs (Table 2) based on menopausal and luminal A status and family history of breast cancer.

			Premenopausal women (n = 1036)		Postmenopausal women (n = 1560)			Luminal A (n = 1828)		Non luminal A (n = 755)			With family history (n = 1089)		Without family history (n = 1404)		
SNP	MA	Controls¶	OR_per-allele_ * [95% CI]	Adjusted *P* *	OR_per-allele_ * [95% CI]	Adjusted *P* *	*P*_het_	OR_per-allele_ * [95% CI]	Adjusted *P* *	OR_per-allele_ * [95% CI]	Adjusted *P* *	*P*_het_	OR_per-allele_ * [95% CI]	Adjusted *P* *	OR_per-allele_ * [95% CI]	Adjusted *P* *	*P*_het_
rs1429142	C	4470	1.49 [1.31–1.68]	6.22E-10	1.17 [1.04–1.31]	7.79E-03	2.00E-03	1.26 [1.14–1.41]	1.62E-05	1.33 [1.15–1.54]	1.30E-04	5.99E-01	1.28 [1.13–1.45]	1.53E-04	1.28 [1.14–1.44]	3.07E-05	9.88E-01
rs1092913	T	4470	1.28 [1.12–1.47]	4.53E-04	1.18 [1.04–1.34]	1.01E-02	2.89E-01	1.17 [1.04–1.32]	9.56E-03	1.30 [1.12–1.53]	9.14E-04	2.12E-01	1.10 [0.95–1.27]	1.98E-01	1.27 [1.12–1.44]	1.69E-04	8.30E-02
rs1981867	T	4470	1.02 [0.91–1.14]	7.19E-01	1.10 [1.00–1.21]	6.02E-02	2.99E-01	1.09 [1.00–1.20]	6.31E-02	1.01 [0.89–1.15]	8.98E-01	2.96E-01	1.10 [0.99–1.23]	8.43E-02	1.06 [0.96–1.18]	2.24E-01	6.03E-01
rs10411161	A	4470	1.13 [0.98–1.31]	8.81E-02	1.11 [0.98–1.27]	9.74E-02	8.31E-01	1.17 [1.04–1.31]	1.17E-02	1.02 [0.86–1.21]	7.91E-01	1.87E-01	1.12 [0.97–1.30]	1.16E-01	1.16 [1.02–1.32]	2.49E-02	6.88E-01
rs3848562	C	4470	1.12 [0.96–1.30]	1.52E-01	1.12 [0.98–1.28]	8.83E-02	9.61E-01	1.17 [1.04–1.32]	1.06E-02	1.00 [0.84–1.20]	9.72E-01	1.23E-01	1.11 [0.95–1.28]	1.85E-01	1.16 [1.01–1.32]	3.21E-02	6.03E-01
rs11878583	C	4470	1.09 [0.94–1.27]	2.50E-01	1.10 [0.97–1.26]	1.44E-01	9.35E-01	1.12 [0.99–1.27]	6.19E-02	1.05 [0.88–1.24]	6.06E-01	4.74E-01	1.10 [0.95–1.27]	2.19E-01	1.13 [0.99–1.29]	6.13E-02	6.89E-01

MA, minor allele; *P*_het_, *P* for heterogeneity.

¶ Common controls used for conducting each of the subgroup analyses mentioned above.

* Adjusted for BMI.

**Table 4:** Subgroup analyses of the six putative breast cancer susceptibility SNPs (Table 2) based on tumor grade and stage.

			High tumor grade (n = 1057)		Low tumor grade (n = 1536)			Tumor stages I-IIIA (n = 2529)		Tumor stage IIIB and IIIC (n = 153)		
SNP	MA	Controls¶	OR_per-allele_ * [95% CI]	Adjusted *P* *	OR_per-allele_ * [95% CI]	Adjusted *P* *	*P*_het_	OR_per-allele_ * [95% CI]	Adjusted *P* *	OR_per-allele_ * [95% CI]	Adjusted *P* *	*P*_het_
rs1429142	C	4470	1.34 [1.18–1.52]	5.04E-06	1.24 [1.11–1.39]	2.41E-04	3.47E-01	1.29 [1.17–1.42]	1.62E-07	1.25 [0.92–1.70]	1.60E-01	8.28E-01
rs1092913	T	4470	1.29 [1.13–1.48]	2.06E-04	1.12 [0.99–1.28]	7.96E-02	8.50E-02	1.24 [1.12–1.38]	3.91E-05	0.80 [0.53–1.20]	2.83E-01	3.80E-02
rs1981867	T	4470	1.11 [0.99–1.23]	7.30E-02	1.05 [0.95–1.16]	3.29E-01	4.51E-01	1.06 [0.98–1.15]	1.34E-01	1.08 [0.83–1.42]	5.69E-01	9.10E-01
rs10411161	A	4470	1.14 [0.99–1.32]	6.68E-02	1.12 [0.98–1.27]	9.16E-02	7.75E-01	1.12 [1.01–1.25]	3.36E-02	1.23 [0.88–1.72]	2.20E-01	5.73E-01
rs3848562	C	4470	1.14 [0.98–1.32]	7.99E-02	1.11 [0.97–1.27]	1.23E-01	7.32E-01	1.13 [1.01–1.26]	3.60E-02	1.16 [0.81–1.64]	4.18E-01	8.56E-01
rs11878583	C	4470	1.12 [0.97–1.30]	1.14E-01	1.08 [0.95–1.24]	2.36E-01	6.46E-01	1.11 [0.99–1.23]	6.83E-02	1.08 [0.75–1.54]	6.88E-01	8.90E-01

MA, minor allele; *P*_het_, *P* for heterogeneity.

¶ Common controls used for conducting each of the subgroup analyses mentioned above.

* Adjusted for BMI.

### Association of SNPs with breast cancer outcomes

Of the 17 SNPs tested for their associations with breast cancer outcomes, 8q24.21-rs13281615 was significantly associated with reduced risk of both RFS (adjusted *P* = 0.001 and adjusted per allele HR and 95% CI = 0.77 [0.65–0.90]) and OS (adjusted *P* = 0.003, adjusted per allele HR and 95% CI = 0.76 [0.64–0.91]) (**Table S3**). The remaining 16 SNPs did not show statistically significant associations with breast cancer outcomes at adjusted *P*<0.05.

## Discussion

In this independent replication study in Canadian women involving 2,750 breast cancer cases and 4,472 healthy controls, we successfully reproduced the associations of ten previously GWAS-identified breast cancer susceptibility loci, indicating the robustness of the consortia identified SNPs with breast cancer. In addition, two of the six putative breast cancer susceptibility SNPs (4q31.22-rs1429142 and 5p15.2-rs1092913) from our previous two-stage GWAS also showed robust associations in an independent set of breast cancer cases and healthy controls (stage 3). After adjusting for BMI, 4q31.22-rs1429142 attained near genome-wide significance level (adjusted *P* = 1.5×10^−7^) (**Table 2**). A major strength of this study is the consideration of BMI, which allowed confirmation that the genetic contributions to breast cancer are independent of one of the major risk factors for breast cancer. An additional strength was our evaluation of the SNP-breast cancer associations as potential prognostic factors for RFS and OS after diagnosis and their relationships with breast cancer clinical and molecular subtypes.

The most notable associations among the ten previously GWAS-identified breast cancer susceptibility loci replicated in this study were with four *FGFR2* SNPs (rs2981579, rs1219648, rs2420946 and rs2981582) and *TNRC9*-rs3803662 (observed *P*<7.0×10^−10^ and adjusted *P*<4.2×10^−8^) (**Table S1**). The magnitude and direction of the associations were similar to those reported in the original GWASs (observed per allele OR ranges: 1.17–1.26) [13]–[16], [18], [19], suggesting the robustness of these associations with breast cancer susceptibility. Further, results from the subgroup analyses were consistent with the previous reports [33]–[35], supporting the hypothesis that *FGFR2* loci (rs1219648, rs2420946 and rs2981582) are associated with increased risk of breast cancer, especially in familial breast cancer cases (*P*_het_<0.02), and associated with the better prognosis luminal A type or estrogen receptor positive breast cancers (*P*_het_<0.001) (**Table S2**) [33]–[35].

Of the six putative breast cancer susceptibility SNPs reported in our previous two-stage GWAS, our independent stage 3 analyses successfully replicated the associations of 4q31.22-rs1429142 and 5p15.2-rs1092913 with increased risk of breast cancer. In the combined analyses, five of the six reported associations from our previous GWAS retained statistical significance, the exception being 16q23.2-rs1981867. These five SNPs should be further tested independently in additional cases and controls to assess their role in breast cancer etiology. When adjusted for BMI, we observed near genome-wide significant association for 4q31.22-rs1429142 (adjusted *P* = 1.7×10^−7^) while 5p15.2-rs1092913 remained statistically significant (adjusted *P* = 1.9×10^−4^). For 4q31.22-rs1429142, there was a substantial increase from the observed ORs (per allele = 1.22, OR_heterozygote_ = 1.26 and OR_homozygote_ = 1.36) to adjusted ORs (per allele = 1.28, OR_heterozygote_ = 1.32 and OR_homozygote_ = 1.52). These results indicate that the 4q31.22-rs1429142-breast cancer association may be linked to the BMI pathway of breast cancer risk elevation. This observation is in contrast to the ten GWAS-identified consortia reported SNP-breast cancer associations, and hence requires replication in independent set of breast cancer cases and controls, probably through collaborative efforts involving large international consortia. Both 4q31.22-rs1429142 and 5p15.2-rs1092913 showed statistically significant associations with breast cancer in subgroups with pre and postmenopausal women, cases with luminal and non-luminal A tumors, with and without family history of breast cancer, low and high tumor grade and operable tumor stage at adjusted *P*<0.05 (**Tables 3** **and** **4**). However, the association of 4q31.22-rs1429142 was stronger in pre than postmenopausal women (*P*_het_ = 0.002), suggesting that 4q31.22-rs1429142-breast cancer association may vary by menopausal status.

Except for 8q24.21-rs13281615, none of the breast cancer susceptibility SNPs, including 4q31.22-rs1429142, showed significant association with breast cancer outcomes. 8q24.21-rs13281615 was significantly associated with better RFS and OS (adjusted *P*<4.5×10^−3^) (**Table S3**). Similar results for 8q24.21-rs13281615 were also observed in another study involving 13,527 invasive breast cancer cases [33]. To our knowledge, this is the second study to identify the potential prognostic value of 8q24.21-rs13281615 and hence this locus merits further investigation. These results provide further evidence supporting the hypothesis that the SNPs with prognostic value are yet to be identified using whole genome approaches and that the SNPs associated with breast cancer susceptibility (etiology) are distinct.

**Figure 1:**
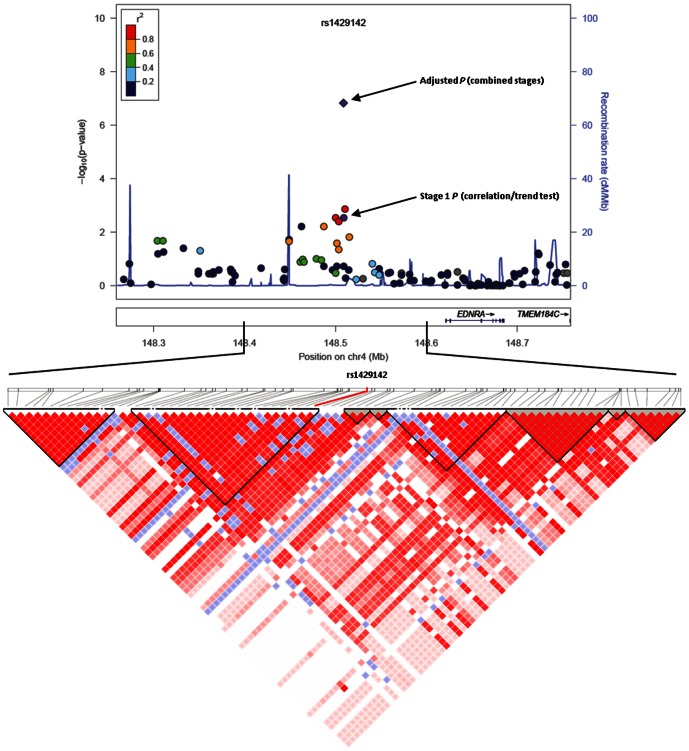
Regional association plot (top panel) for 4q31.22-rs1429142 using LocusZoom [48], with the association *P* values (−log10 *P*) on the y-axis and the chromosomal position (hg18) on x-axis. The association of 4q31.22-rs1429142 in stage 1 is shown in purple circle while association in combined stages (1+2+3) after adjusting for BMI is shown in purple diamond. Pair-wise linkage disequilibrium (LD) of 4q31.22-rs1429142 with adjacent SNPs are measured by *r^2^* values (from HapMap Phase II CEU data) and represented by the color of each circle. Neighbouring Refseq genes are shown below the plot. LD profiles (bottom panel) among SNPs located within 100 kb up and downstream of the 4q31.22-rs1429142, using HapMap Phase II CEU data are presented.

4q31.22-rs1429142 is located in a gene desert, with the closest gene endothein receptor type A (*EDNRA*) (**Figure 1**) located ∼112 kb downstream of the SNP. *EDNRA* gene encoded protein is a cell surface bound receptor involved in several fundamental cellular processes by interacting with endothelins (widely expressed cytokines in various tissues) [36]. SNPs in or near the *EDNRA* gene have been associated with intracranial aneurysm risk [37], hypertension [38] and migraines [39]. This SNP is ∼112 kb away from the *EDNRA* gene locus and we therefore queried the SCAN database [40], which uses HapMap human lymphoblastoid cell lines to identify putative expression quantitative trait loci. We found that 4q31.22-rs1429142 is associated with differential expression of five other genes (quantitative transmission disequilibrium test *P*<0.0001, implemented in the SCAN database) involved in at least one type of cancer – *i.e.*, kinesin family member 3B (*KIF3B*) [41], paxillin (*PXN*) [42], general transcription factor IIA, 12 kDa (*GTF2A2*) [43], *PTPRF* interacting protein, binding protein (liprin beta 2) (*PPFIBP2*) [44] and tumor protein p63 regulated 1-like (*TPRG1L*) [45]. However, the allele of 4q31.22-rs1429142 responsible for these is unknown and future fine mapping studies to identify the causal variant and to investigate its allele specific effects are warranted.

5p15.2-rs1092913 is also located in a gene desert. The closest gene is rhophilin associated tail protein 1-like (*ROP1NL*) located ∼2.5 kb upstream of the polymorphism. *ROP1NL* gene encodes a sperm protein, which interacts with A-kinase anchoring protein. Recently, an independent study (n = 4,325 cases and controls) also showed significant association of 5p15.2-rs1092913 with breast cancer risk in estrogen receptor positive breast cancer of Korean ethnicity, suggesting the potential generalizability of this SNP-breast cancer association in the Korean population [46]. Furthermore, a meta-analysis of two GWASs also found multiple SNPs within the *ROP1NL* locus associated with the phenotype of BMI at 5p15.2, suggesting that this region is important for both breast cancer susceptibility and BMI [47].

In summary, our study not only provided supportive evidence for the robustness of the breast cancer susceptibility SNPs previously identified by consortia, but also identified a new locus at 4q31.22-rs1429142 for contributing to breast cancer susceptibility, lending credence to the continued research efforts in search of common variants for breast cancer.

## Supporting Information

Table S1 Associations of the previously identified (consortia SNPs) breast cancer susceptibility loci in the current study.
(XLSX)

Table S2 Subgroup analysis of the 11 previously GWAS-identified SNPs based on menopausal and luminal A status, family history of breast cancer, tumor grade and stage.
(XLSX)

Table S3 Association of the 17 SNPs with breast cancer outcomes.
(XLSX)
